# In Vitro Detoxification of Aflatoxin B_1_, Deoxynivalenol, Fumonisins, T-2 Toxin and Zearalenone by Probiotic Bacteria from Genus *Lactobacillus* and *Saccharomyces cerevisiae* Yeast

**DOI:** 10.1007/s12602-018-9512-x

**Published:** 2019-02-05

**Authors:** Agnieszka Chlebicz, Katarzyna Śliżewska

**Affiliations:** Institute of Fermentation Technology and Microbiology, Department of Biotechnology and Food Sciences, Lodz University of Technology, Wólczańska 171/173, 90-924 Łódź, Poland

**Keywords:** Mycotoxins, Detoxification, Probiotics, *Lactobacillus*, *Saccharomyces cerevisiae*

## Abstract

The aim of the following research was to determine the detoxification properties of probiotic *Lactobacillus* sp. bacteria (12 strains) and *S. cerevisiae* yeast (6 strains) towards mycotoxins, such as aflatoxin B_1_, deoxynivalenol, fumonisins, T-2 toxin and zearalenone, which pose as frequent feed contamination. The experiment involved analysing changes in concentration of mycotoxins in PBS solutions, after 6, 12 and 24 h of incubation with monocultures of tested microorganisms, measured by high-performance liquid chromatography (HPLC). We found that all strains detoxified the mycotoxins, with the highest reduction in concentration observed for the fumonisin B_1_ and B_2_ mixture, ranging between 62 and 77% for bacterial strains and 67–74% for yeast. By contrast, deoxynivalenol was the most resistant mycotoxin: its concentration was reduced by 19–39% by *Lactobacillus* sp. strains and 22–43% by yeast after 24 h of incubation. High detoxification rates for aflatoxin B_1_, T-2 toxin and zearalenone were also observed, with concentration reduced on average by 60%, 61% and 57% by *Lactobacillus*, respectively, and 65%, 69% and 52% by yeast, respectively. The greatest extent of reduction in the concentration for all mycotoxins was observed after 6 h of incubation; however, a decrease in concentration was noted even after 24 h of incubation. Thus, the tested microorganisms can potentially be used as additives to decrease the concentrations of toxins in animal feed.

## Introduction

Mycotoxins are secondary metabolites with low molecular mass (~ 700 Da), which are synthesised by filamentous fungi, belonging mostly to the *Ascomycota* phylum. The most common source of food and feed contamination are mycotoxins produced by the fungi *Aspergillus*, *Penicillium* and *Fusarium* genera [1–3]. Other mycotoxin-producing fungi include *Alternaria*, *Chaetomium*, *Cladosporium*, *Claviceps*, *Diplodia*, *Myrothecium*, *Monascus*, *Phoma*, *Phomopsis*, *Pithomyces*, *Trichoderma* and *Stachybotrys* [4]. Aflatoxins (AF), ochratoxin (OT), trichotecens, including deoxynivalenol (DON) and T-2 toxin (T-2), as well as fumonisins (FUM), along with zearalenone (ZEN) are the most prevalent mycotoxin-related contamination found in fodder [5, 6]. AFB_1_ is most frequently found in feed in the European Union (> 98% of tested samples); however, DON (~ 90% of tested samples) and ZEN (~ 70% of tested samples) are often detected as well. The presence of FUM and OTA, on the other hand, are more sparsely observed [7].

Plants are contaminated with mycotoxins, synthesised by filamentous fungi, most frequently at the time of cultivation in the fields (e.g. mycotoxin produced mostly by *Fusarium* sp.). Likewise, under favourable growth conditions of temperature and humidity, mycotoxin-producing fungi, such as *Aspergillus* sp. and *Penicillium* sp., are also found in food and feed that are stored [2, 8, 9]. In stored grains, moisture content within 16–30%, high temperature reaching 25–30 °C, and high relative air humidity (80–100%) are conditions that stimulate growth of filamentous fungi and mycotoxin production [10]. The concentration of toxins in input materials (e.g. corn, grass, clover) is not reduced to a sufficient degree while they are being processed into feed, as these metabolites are resistant to high and low temperatures, even after long storage period [9, 11]. Therefore, these toxins constitute a threat, as they can enter the human food chain through products such as milk, meat or eggs [2]. Furthermore, humans are exposed to mycotoxin-related intoxications while consuming foods of plant origin, for instance hazelnuts, almonds, grains and fruits [8]. Therefore, European Union legislation specifies tolerable daily intake (TDI) for a variety of mycotoxins, in addition to providing guidance values for their concentrations in animal feedstuffs (Table 1) [18].

**Table 1:** Optimal conditions for mycotoxins production, TDI in food products and guidance value in feedstuff in European Union [12–17]

Mycotoxin	TDI in food (μg/kg)*	Guidance value in feedstuff (12% moisture) (mg/kg)**	Optimum temperature for mycotoxin production (°C)	Optimum water activity for mycotoxin production
AF***	0.025–15.0	0.005–0.05	33	0.99
DON	200–1750	0.9–12	26–30	0.995
FUM****	200–2000	5–60	15–30	0.9–0.995
OTA	0.50–10.0	0.05–0.25	25–30	0.98
T-2/HT-2	15–1000	0.25–2	20–30	0.98–0.995
ZEA	20–200	0.1–3	25	0.96

*Depending on a product (e.g. nuts intended for processing or direct consumption, raw grains, milk, dried fruits, spices, infants’ formulas, processed foods based on cereals, wine, coffee, etc.)

**With a distinction between feed materials and complementary and complete feed mixtures

***Depending on form

****Sum of FB_1_ and FB_2_

The regulation (EU) 2017/625 of the European Parliament and of the Council of 15 March 2017, regarding official controls and other official activities performed to ensure the application of food and feed law, rules on animal health and welfare, plant health and plant protection products will come into effect on 14th December 2019. According to this regulation, Member States are obliged to establish multiannual plans and carry out food and feed controls, to ensure safety in the agri-food chain, as well as animal welfare and health, thereby providing safe food [19].

The process of detoxification or mycotoxin removal is complicated, especially because of heat stability of these compounds, and their breakdown into toxic products. Nevertheless, detoxification may be accomplished by application of the following methods: physical (cooking, baking, microwave heating, radiation, etc.), chemical (use of ammonia, hydrochloric acid, salicylic, sulfamide, sulfosalicylic, anthranilic, benzoic, boric, oxalic or propionic acid), and biological [20]. For biological detoxification, plant extracts, such as piperine, lutein, carotenoids or essential oils, as well as enzymes, such as AF decomposing peroxidase or laccase, and FUM degradative carboxylesterase or aminotransferase are used. Microorganisms that are capable of degrading mycotoxins to less toxic metabolites, and the proper treatment of food or feed by fermentation are also used for biological detoxification [9, 20–23]. In comparison to physical and chemical methods, biological detoxification is more efficient, specific and safer for the environment [22].

In 1998, El-Nazami performed pioneering in vitro studies on the binding properties of mycotoxin by lactic acid bacteria, which has initiated a systematic search for microorganisms having specific abilities to adsorb mycotoxins [24]. Among other microbes identified for biological detoxification purposes, probiotic microorganisms, defined by FAO/WHO (2002) as ‘live microorganisms, which when administered in adequate amounts confer a health benefit on the host’, were identified as microbes that bind and adsorb mycotoxins [25]. Probiotic microorganisms, such as bacteria belonging to genera *Lactobacillus*, *Bifidobacterium*, as well as *Enterococcus faecium*, and the yeasts *Saccharomyces cerevisiae* and *Saccharomyces boulardii* have been shown to have mycotoxin detoxification properties [26].

The aim of our study was to determine detoxification properties of probiotic strains of *Lactobacillus* sp. and *S. cerevisiae* towards mycotoxins, which often contaminate feed for livestock animals. This study was part of one of the stages of strain selection for designing synbiotic preparations for poultry and swine.

## Materials and Methods

### Biological Materials

The biological material included potential probiotic bacteria of *Lactobacillus* genus and strains of the yeast *S. cerevisiae*, which are deposited in the Łódź Collection of Pure Cultures 105 of Institute of Fermentation Technology and Microbiology at Technical University of Łódź (Table 2). Five *Lactobacillus* (*rhamnosus* ŁOCK 1087, *paracasei* ŁOCK 1091, *reuteri* ŁOCK 1092, *plantarum* ŁOCK 0860, *pentosus* ŁOCK 1094) and one *S. cerevisiae* (ŁOCK 0119) strain have been documented in patent application no. 422603 [27].

**Table 2:** Strains whose mycotoxin detoxification properties has been studied

Microorganism		Collection number	Source of isolation
Bacteria	*Lact. brevis*	ŁOCK 1093	Plant silages
	*Lact. casei*	ŁOCK 0911	Milk fermented beverages
	*Lact. casei*	ŁOCK 0915	Milk fermented beverages
	*Lact. paracasei*	ŁOCK 1091	Caecal content of sow
	*Lact. pentosus*	ŁOCK 1094	Broiler chicken dung
	*Lact. plantarum*	ŁOCK 0860	Plant silages
	*Lact. plantarum*	ŁOCK 0862	Plant silages
	*Lact. reuteri*	ŁOCK 1092	Piglet caecal content
	*Lact. reuteri*	ŁOCK 1096	Winear pig’s intestinal content
	*Lact. rhamnosus*	ŁOCK 1087	Turkey dung
	*Lact. rhamnosus*	ŁOCK 1088	Broiler chicken’s intestinal content
	*Lact. rhamnosus*	ŁOCK 1089	Broiler chicken’s intestinal content
Yeast	*S. cerevisiae*	ŁOCK 0068	Forage
	*S. cerevisiae*	ŁOCK 0113	Distillers’ yeast, potato with grain
	*S. cerevisiae*	ŁOCK 0119	Distillers’ yeast, grain
	*S. cerevisiae*	ŁOCK 0137	Baker’s yeast
	*S. cerevisiae*	ŁOCK 0140	Baker’s yeast
	*S. cerevisiae*	ŁOCK 0142	Baker’s yeast

The detoxification activity of bacteria and yeast was determined against five mycotoxins, namely aflatoxin B1, fumonisin mixture of fumonisin B1 and B2 (FUM), T-2 toxin, zearalenone and deoxynivalenol (Table 3). Mycotoxins were suspended in PBS buffer (Calbiochem®, Germany), and solutions of 100 μg/ml were prepared.

**Table 3:** Mycotoxins, that were detoxified by selected strains of potentially probiotic microorganisms (Sigma-Aldrich, available online on https://www.sigmaaldrich.com/, accessed on 21 March 2018 [28–33])

Mycotoxin	Chemical structure	Producer, catalogue number	Producer, catalogue number of HPLC sample
AFB_1_		Sigma, A6636	Supelco, 46323-u
DON		Sigma, 32943	Supelco, CRM46911
FUM mixture	Fumonisin B_1_	Sigma, 34143	Fumonisin B_1_ – Sigma, 34139
	Fumonisin B_2_		Fumonisin B_2_ – Sigma, 34142
T-2		Sigma, T4887	Sigma, 34071
ZEA		Sigma, Z2125	Supelco, CRM46916

### Bacterial and Yeast Strain Cultivation and Sample Preparation

*Lactobacillus* sp. were cultivated in de Man, Rogosa, and Sharpe (MRS) broth (Merck, Germany) at 37 °C, while *S. cerevisiae* was grown at 30 °C, in yeast extract–peptone–glucose broth (YPG Broth, Merck, Germany). Both bacteria and yeast cultures were grown in normal oxygen conditions for 24 h. After 24 h of incubation, monocultures of the analysed strains, in three repetitions, were centrifuged at relative centrifugal force (RCF) 3468×*g* for 10 min (Centrifuge MPW-251; MPW, Poland). Subsequently, the supernatants were removed and the bacteria and yeast biomass were washed three times with PBS buffer to remove any residual culture medium. The cell pellets were again centrifuged under the same conditions. Ten millilitres of mycotoxin solutions were added to the prepared samples, with a defined concentration of 100 μg/ml for each mycotoxin. These samples were further incubated in normal oxygen conditions for 24 h at 37 °C or 30 °C for lactic acid bacteria and yeast, respectively. After 6, 12 and 24 h of incubation, 2 ml of each sample was collected, centrifuged at RCF of 3468×*g* for 10 min, and the supernatants were filtered with PTFE syringe filters with 0.22-μm-diameter pores (Millex-GS, Millipore, USA). As a positive control sample, a solution of analysed mycotoxin in PBS was used, and bacterial or yeast suspension served as negative control sample.

### HPLC Analysis

The prepared samples were subjected to high-performance liquid chromatography (HPLC) analysis, the parameters of which are presented in Table 4. Analysis was performed as previously described by El-Nazami et al. [24], with modifications. For this purpose, Surveyor liquid chromatography (Thermo Scientific, USA) was used with a 250 × 4.6 mm size ACE 5 C18 column (Advanced Chromatography Technologies (ACT), Scotland). Mycotoxins were identified by comparing the retention times of the peak with standard solutions. The mycotoxin concentrations were determined by correlation of peak area of the samples with the standard curves, obtained by HPLC analysis of standard solutions.

**Table 4:** HPLC analysis parameters

Parameter	Mycotoxins				
	AFB_1_	DON	FUM	T-2	ZEN
Column heating	–	30	–	–	–
Mobile phase	Water/acetonitrile/methanol (60:30:10)	Water/acetonitrile (90:10)	Gradient methanol/water (70:30 and 80:20)	Methanol/water (60:40)	Methanol/water (70:30)
Fluorescent detector λ (nm) (excitation and emission)	360 and 420	–	490 and 450	381 and 470	280 and 460
UV detector λ (nm)	–	218	–	–	–
Flow (ml/min)	1	1	1	1	1

### Statistical Analysis

The results presented here constitute the arithmetic mean of values from three repetitions, with standard deviation. All statistical analyses were carried out using the one-way ANOVA test, with a significance level of *p* < 0.05 (Origin 6.1 program, OriginLab). A comparative Duncan test was carried out at a significance level of *p* > 0.05 (STATISTICA 10, StatSoft).

## Results

### AFB_1_ Microbial Detoxification

Bacteria belonging to the *Lactobacillus* genus were characterised by their diverse ability to detoxify aflatoxin B_1_. In merely after 6 h of incubation, a statistically significant reduction of AFB_1_ concentration was noticed, ranging from 35.33 to 79.65 μg/ml (20–65% reduction, 49% on average) compared to the initial mycotoxin concentration of 100 μg/ml. In subsequent hours, further reduction in AFB_1_ concentration was observed. After 24 h of incubation, the concentration of mycotoxin in the samples was 28.96–55.80 μg/ml (mean, 40.43 μg/ml). Therefore, there was a reduction of between 44 and 71% (mean, 60%) compared to the initial concentration of the mycotoxin.

*S. cerevisiae* showed detoxification activity similar to that of analysed strains of *Lactobacillus* sp. After 6 h of incubation, AFB_1_ concentrations were statistically significantly reduced by 47–66% (average 58%) and ranged from 33.64 to 53.27 μg/ml. In subsequent hours of incubation, the concentration of the mycotoxin further decreased, and after 24 h, the mycotoxin concentrations were 32.48–4.45 μg/ml (average reduction of AFB1 by 65%) (Table 5).

**Table 5:** Reduction of AFB_1_ concentration by *Lactobacillus* and *S. cerevisiae* strains

	Strain	Time (h)			
		0	6	12	24
		Concentration ± SD (μg/ml) (decrease (%))			
*Lactobacillus*	*brevis* ŁOCK 1093	100 ^A^	39.56 ± 0.99 ^B^ (60)	37.13 ± 2.04 ^B^ (63)	32.70 ± 0.59 ^C^ (67)
	*casei* ŁOCK 0911		51.30 ± 2.04 ^B^ (49)	49.92 ± 1.36 ^B^ (50)	45.13 ± 0.38 ^C^ (55)
	*casei* ŁOCK 0915		67.18 ± 0.63 ^B^ (33)	58.98 ± 1.27 ^C^ (41)	55.80 ± 1.51 ^D^ (44)
	*paracasei* ŁOCK 1091		57.44 ± 1.61 ^B^ (43)	48.23 ± 1.27 ^C^ (52)	42.21 ± 1.52 ^D^ (58)
	*pentosus* ŁOCK 1094		44.70 ± 0.41 ^B^ (55)	38.73 ± 1.03 ^B^ (61)	28.96 ± 0.58 ^B^ (71)
	*plantarum* ŁOCK 0860		47.93 ± 1.01 ^B^ (52)	45.88 ± 0.88 ^B^ (54)	40.62 ± 0.50 ^C^ (59)
	*plantarum* ŁOCK 0862		35.33 ± 0.95 ^B^ (65)	34.82 ± 0.47 ^B^ (65)	34.30 ± 1.06 ^B^ (66)
	*reuteri* ŁOCK 1092		45.19 ± 0.97 ^B^ (55)	44.98 ± 0.60 ^B^ (55)	43.79 ± 1.81 ^B^ (56)
	*reuteri* ŁOCK 1096		40.79 ± 0.74 ^B^ (59)	38.41 ± 1.08 ^C^ (62)	36.02 ± 0.57 ^D^ (64)
	*rhamnosus* ŁOCK 1087		41.42 ± 2.34 ^B^ (59)	40.32 ± 0.34 ^B^ (60)	40.19 ± 1.48 ^B^ (60)
	*rhamnosus* ŁOCK 1088		79.65 ± 0.96 ^B^ (20)	61.04 ± 0.88 ^C^ (39)	44.83 ± 1.49 ^D^ (55)
	*rhamnosus* ŁOCK 1089		56.24 ± 0.98 ^B^ (44)	50.43 ± 0.46 ^C^ (50)	40.61 ± 1.58 ^D^ (59)
	Average concentration (μg/ml) (decrease (%))		50.56 (49)	45.74 (54)	40.43 (60)
*S. cerevisiae*	ŁOCK 0068	100 A	46.79 ± 0.62 ^B^ (53)	44.43 ± 1.35 ^B^ (56)	38.07 ± 1.35 ^C^ (62)
	ŁOCK 0113		53.27 ± 0.69 ^B^ (47)	48.93 ± 1.75 ^C^ (51)	41.45 ± 0.63 ^D^ (59)
	ŁOCK 0119		42.25 ± 0.73 ^B^ (58)	34.12 ± 1.05 ^C^ (66)	31.48 ± 1.05 ^D^ (69)
	ŁOCK 0137		34.99 ± 1.85 ^B^ (65)	34.02 ± 2.30 ^B^ (66)	32.70 ± 0.79 ^B^ (67)
	ŁOCK 0140		41.55 ± 1.62 ^B^ (58)	39.58 ± 1.42 ^B^ (60)	35.78 ± 1.39 ^C^ (64)
	ŁOCK 0142		33.64 ± 0.48 ^B^ (66)	33.00 ± 1.41 ^B,C^ (67)	32.48 ± 0.38 ^C^ (68)
	Average concentration (μg/ml) (decrease (%))		42.08 (58)	39.01 (61)	35.33 (65)

*Values labelled by different capital letters were significantly different per analysed strain (*p* < 0.05)

### DON Microbial Detoxification

Deoxynivalenol (DON) concentrations were significantly reduced after 6 h of incubation in the presence of the bacteria monoculture, varying between 78.39 and 94.24 μg/ml (mean, 84.30 μg/ml), indicating that these bacteria have the ability to decrease DON concentration by an average of 16%. Further decrease in DON concentration in the subsequent incubation hours was also observed. After 24 h, DON concentrations ranged between 60.63 and 80.72 μg/ml (mean, 70.45 μg/ml), thereby showing a reduction of 19–39% (mean, 30%).

*S. cerevisiae* strains used in the analysis also demonstrated the ability to reduce the concentration of DON in suspension. After 6 h, significant reductions of DON concentrations were observed, ranging between 12 and 22% (mean, 18%) of the initial concentration of DON. After a further 6 h, the concentrations were 65.57–80.81 μg/ml (mean, 73.20 μg/ml). After 24 h of incubation, the DON concentrations were reduced by 22–40% (mean, 33%) relative to the initial concentration of mycotoxin (Table 6).

**Table 6:** Reduction of DON concentration by *Lactobacillus* and *S. cerevisiae* strains

	Strain	Time (h)			
		0	6	12	24
		Concentration ± SD (μg/ml) (decrease (%))			
*Lactobacillus*	*brevis* ŁOCK 1093	100 ^A^	94.24 ± 0.77 ^B^ (6)	87.52 ± 0.95 ^C^ (12)	80.72 ± 0.33 ^D^ (19)
	*casei* ŁOCK 0911		92.06 ± 1.10 ^B^ (8)	84.06 ± 1.01 ^C^ (16)	72.49 ± 0.67 ^D^ (28)
	*casei* ŁOCK 0915		88.73 ± 1.85 ^B^ (11)	81.93 ± 0.75 ^C^ (18)	78.06 ± 1.20 ^D^ (22)
	*paracasei* ŁOCK 1091		84.44 ± 1.75 ^B^ (16)	73.75 ± 3.09 ^C^ (26)	67.30 ± 1.46 ^D^ (33)
	*pentosus* ŁOCK 1094		78.53 ± 1.19 ^B^(21)	72.49 ± 1.88 ^C^ (27)	66.82 ± 0.65 ^D^ (33)
	*plantarum* ŁOCK 0860		80.34 ± 0.49 ^B^ (20)	74.12 ± 0.83 ^C^ (26)	70.25 ± 1.01 ^D^ (30)
	*plantarum* ŁOCK 0862		83.78 ± 0.20 ^B^ (16)	78.34 ± 1.27 ^C^ (22)	74.53 ± 1.04 ^D^ (25)
	*reuteri* ŁOCK 1092		79.92 ± 1.02 ^B^ (20)	69.05 ± 0.44 ^C^ (31)	60.63 ± 0.59 ^D^ (39)
	*reuteri* ŁOCK 1096		78.36 ± 0.30 ^B^ (22)	70.58 ± 1.38 ^C^ (29)	61.31 ± 1.45 ^D^ (39)
	*rhamnosus* ŁOCK 1087		87.85 ± 2.94 ^B^ (12)	79.04 ± 1.13 ^C^ (21)	75.05 ± 1.43 ^D^ (25)
	*rhamnosus* ŁOCK 1088		83.04 ± 2.78 ^B^ (17)	78.14 ± 0.86 ^C^ (22)	73.74 ± 0.30 ^D^ (26)
	*rhamnosus* ŁOCK 1089		80.35 ± 0.49 ^B^ (20)	74.01 ± 2.61 ^C^ (26)	64.55 ± 0.70 ^D^ (35)
	Average concentration (μg/ml) (decrease (%))		84.30 (16)	76.92 (23)	70.45 (30)
*S. cerevisiae*	ŁOCK 0068	100 A	77.68 ± 1.53 ^B^ (22)	70.62 ± 1.17 ^C^ (29)	64.57 ± 0.57 ^D^ (35)
	ŁOCK 0113		88.32 ± 0.88 ^B^ (12)	80.81 ± 1.45 ^C^ (19)	78.03 ± 0.82 ^D^ (22)
	ŁOCK 0119		80.87 ± 0.85 ^B^ (19)	65.57 ± 1.52 ^C^ (34)	57.50 ± 0.83 ^D^ (43)
	ŁOCK 0137		84.16 ± 1.40 ^B^ (16)	80.41 ± 2.27 ^B^ (20)	76.10 ± 1.32 ^C^ (24)
	ŁOCK 0140		80.64 ± 1.71 ^B^ (19)	71.97 ± 2.04 ^C^ (20)	63.07 ± 1.49 ^D^ (37)
	ŁOCK 0142		80.28 ± 2.07 ^B^ (20)	69.80 ± 1.61 ^C^ (30)	60.91 ± 1.24 ^D^ (39)
	Average concentration (μg/ml) (decrease (%))		81.99 (18)	73.20 (27)	66.70 (33)

*Values labelled by different capital letters were significantly different per analysed strain (*p* < 0.05)

### FUM Microbial Detoxification

Bacteria belonging to *Lactobacillus* genus detoxified the mixture of fumonisin B_1_ (FB_1_) and B_2_ (FB_2_) mycotoxins (FUMs). After 6 h of incubation, the FUM concentration was reduced by 36–64% (mean, 51%) compared to the initial concentration of the mycotoxin mixture. Subsequent incubation resulted in a further significant decrease in FUM concentration, which after 24 h of incubation was reduced by an average of 70% and ranged from 23.08 to 38.42 μg/ml (mean, 29.53 μg/ml).

*S. cerevisiae* also reduced the concentrations of FUM, significantly reducing them by 29–60% (mean, 53%) to 40.15–70.57 μg/ml (mean, 47.19 μg/ml). In subsequent hours of incubation, the concentrations of mycotoxin further declined. Finally, after 24 h, the FUM concentration ranged between 25.53 and 32.57 μg/ml, demonstrating an average reduction of 72% of the initial concentration (Table 7).

**Table 7:** Reduction of FUM concentration by *Lactobacillus* and *S. cerevisiae* strains

	Strain	Time (h)			
		0	6	12	24
		Concentration ± SD (μg/ml) (decrease (%))			
*Lactobacillus*	*brevis* ŁOCK 1093	100 ^A^	50.14 ± 1.26 ^B^ (50)	43.87 ± 1.01 ^C^ (56)	34.27 ± 1.26 ^D^ (66)
	*casei* ŁOCK 0911		47.80 ± 3.20 ^B^ (52)	40.63 ± 0.46 ^C^ (59)	36.32 ± 3.27 ^C^ (64)
	*casei* ŁOCK 0915		48.52 ± 3.21 ^B^ (51)	41.98 ± 1.81 ^C^ (58)	33.50 ± 1.11 ^D^ (67)
	*paracasei* ŁOCK 1091		55.98 ± 3.55 ^B^ (44)	45.56 ± 1.57 ^C^ (54)	38.42 ± 1.39 ^D^ (62)
	*pentosus* ŁOCK 1094		45.24 ± 0.41 ^B^ (55)	38.61 ± 1.02 ^C^ (61)	29.61 ± 1.04 ^D^ (70)
	*plantarum* ŁOCK 0860		35.52 ± 1.92 ^B^ (64)	30.88 ± 0.69 ^C^ (70)	25.11 ± 1.67 ^D^ (75)
	*plantarum* ŁOCK 0862		38.72 ± 1.04 ^B^ (61)	30.54 ± 0.08 ^C^ (69)	23.19 ± 1.60 ^D^ (77)
	*reuteri* ŁOCK 1092		42.64 ± 1.18 ^B^ (57)	37.38 ± 1.34 ^C^ (63)	27.23 ± 1.17 ^D^ (73)
	*reuteri* ŁOCK 1096		41.83 ± 1.20 ^B^ (58)	34.62 ± 2.32 ^C^ (65)	28.22 ± 1.05 ^D^ (72)
	*rhamnosus* ŁOCK 1087		56.53 ± 2.13 ^B^ (43)	45.39 ± 0.36 ^C^ (55)	24.00 ± 0.69 ^D^ (76)
	*rhamnosus* ŁOCK 1088		63.64 ± 1.98 ^B^ (36)	53.87 ± 1.27 ^C^ (46)	31.43 ± 0.95 ^D^ (69)
	*rhamnosus* ŁOCK 1089		58.75 ± 1.35 ^B^ (41)	38.67 ± 1.08 ^C^ (61)	23.08 ± 0.08 ^D^ (77)
	Average concentration (μg/ml) (decrease (%))		48.77 (51)	40.17 (60)	29.53 (70)
*S. cerevisiae*	ŁOCK 0068	100 ^A^	70.57 ± 0.64 ^B^ (29)	56.03 ± 1.13 ^C^ (44)	32.57 ± 2.21 ^D^ (67)
	ŁOCK 0113		48.73 ± 0.32 ^B^ (51)	40.12 ± 1.08 ^C^ (60)	27.12 ± 1.06 ^D^ (73)
	ŁOCK 0119		54.98 ± 1.73 ^B^ (45)	43.91 ± 1.15 ^C^ (56)	26.96 ± 1.00 ^D^ (73)
	ŁOCK 0137		40.15 ± 1.07 ^B^ (50)	34.38 ± 2.11 ^C^ (66)	26.88 ± 1.54 ^D^ (73)
	ŁOCK 0140		58.13 ± 0.86 ^B^ (42)	47.09 ± 1.84 ^C^ (53)	29.29 ± 1.50 ^D^ (71)
	ŁOCK 0142		44.29 ± 0.97 ^B^ (56)	37.41 ± 0.76 ^C^ (63)	25.53 ± 0.65 ^D^ (74)
	Average concentration (μg/ml) (decrease (%))		47.19 (53)	43.16 (57)	28.06 (72)

*Values labelled by different capital letters were significantly different per analysed strain (*p* < 0.05)

### T-2 Microbial Detoxification

After 6 h of incubation, significant reduction of T-2 toxin concentrations by monocultures of analysed *Lactobacillus* sp. strains were observed ranging between 48.25 and 73.32 μg/ml (mean, 57.36 μg/ml). Continued incubation caused a further decrease in T-2 concentration, as a result of which after 24 h of incubation the concentrations of mycotoxin were 31.09–50.10 μg/ml (mean, 39.01 μg/ml). This shift in concentration values indicated a reduction of 50–69% (mean, 61%) in relation to the initial quantity of T-2.

*S. cerevisiae* strains subjected to the analysis were characterised by diverse T-2 detoxification activity. After 6-h incubation, a statistically significant decrease in the concentration of T-2 to level of 46.92–54.98 μg/ml (average reduction of 49% of initial concentration) was observed. In subsequent hours of incubation, T-2 concentration further decreased. The concentration of the mycotoxin after 24 h of incubation reduced by 60–63% (mean, 61%) of the initial concentration and ranged between 37.36 and 40.40 μg/ml (mean, 38.68 μg/ml) (Table 8).

**Table 8:** Reduction of T-2 concentration by *Lactobacillus* and *S. cerevisiae* strains

	Strain	Time (h)			
		0	6	12	24
		Concentration ± SD (μg/ml) (decrease (%))			
*Lactobacillus*	*brevis* ŁOCK 1093	100 ^A^	73.32 ± 3.18 ^B^ (27)	60.74 ± 0.41 ^C^ (39)	50.10 ± 1.32 ^D^ (50)
	*casei* ŁOCK 0911		54.11 ± 1.21 ^B^ (46)	45.04 ± 1.59 ^C^ (55)	36.83 ± 0.46 ^D^ (63)
	*casei* ŁOCK 0915		51.86 ± 3.06 ^B^ (48)	43.24 ± 0.96 ^C^ (57)	35.90 ± 0.48 ^D^ (64)
	*paracasei* ŁOCK 1091		60.17 ± 1.62 ^B^ (40)	53.57 ± 1.83 ^C^ (46)	48.24 ± 0.83 ^D^ (52)
	*pentosus* ŁOCK 1094		59.06 ± 2.45 ^B^ (41)	43.47 ± 1.43 ^C^ (57)	39.24 ± 1.41 ^D^ (61)
	*plantarum* ŁOCK 0860		48.62 ± 0.03 ^B^ (52)	39.08 ± 0.17 ^C^ (61)	31.46 ± 0.71 ^D^ (69)
	*plantarum* ŁOCK 0862		61.76 ± 3.04 ^B^ (38)	48.28 ± 1.48 ^C^ (52)	42.71 ± 1.07 ^D^ (57)
	*reuteri* ŁOCK 1092		53.86 ± 3.18 ^B^ (46)	39.99 ± 1.20 ^C^ (60)	31.09 ± 0.69 ^D^ (69)
	*reuteri* ŁOCK 1096		55.93 ± 3.57 ^B^ (44)	42.39 ± 1.11 ^C^ (58)	36.41 ± 0.72 ^D^ (64)
	*rhamnosus* ŁOCK 1087		48.25 ± 1.24 ^B^ (52)	38.18 ± 1.37 ^C^ (62)	26.76 ± 1.30 ^D^ (73)
	*rhamnosus* ŁOCK 1088		58.30 ± 4.13 ^B^ (52)	49.88 ± 1.20 ^C^ (51)	38.10 ± 1.42 ^D^ (62)
	*rhamnosus* ŁOCK 1089		63.08 ± 1.45 ^B^ (37)	51.25 ± 1.99 ^C^ (49)	45.78 ± 0.56 ^D^ (54)
	Average concentration (μg/ml) (decrease (%))		57.36 (43)	46.66 (54)	39.01 (61)
*S. cerevisiae*	ŁOCK 0068	100 ^A^	53.66 ± 1.37 ^B^ (46)	38.96 ± 2.43 ^C^ (61)	31.83 ± 1.64 ^D^ (68)
	ŁOCK 0113		47.78 ± 1.34 ^B^ (52)	40.04 ± 1.44 ^C^ (60)	32.25 ± 0.77 ^D^ (68)
	ŁOCK 0119		46.92 ± 2.32 ^B^ (53)	37.57 ± 0.53 ^C^ (62)	31.06 ± 1.23 ^D^ (69)
	ŁOCK 0137		51.51 ± 1.34 ^B^ (49)	37.73 ± 1.30 ^C^ (62)	27.71 ± 1.40 ^D^ (72)
	ŁOCK 0140		54.98 ± 1.56 ^B^ (45)	40.44 ± 1.75 ^C^ (60)	32.62 ± 0.63 ^D^ (67)
	ŁOCK 0142		52.23 ± 0.92 ^B^ (48)	37.36 ± 0.67 ^C^ (62)	29.48 ± 0.86 ^D^ (71)
	Average concentration (μg/ml) (decrease (%))		51.18 (49)	38.68 (61)	30.54 (69)

*Values labelled by different capital letters were significantly different per analysed strain (*p* < 0.05)

### ZEN Detoxification

*Lactobacillus* sp. showed varied ability for zearalenone (ZEN) detoxification. After only 6 h of incubation, a statistically significant reduction of ZEN concentration was observed, which ranged between 28 and 59% (mean, 43%) relative to the initial mycotoxin concentration of 100 μg/ml. In subsequent hours of incubation, a further reduction in the mycotoxin concentration was noted. After 24 h of incubation, the concentration of ZEN was 27.39–60.05 μg/ml (mean, 40.43 μg/ml). Therefore, a reduction of 40–73% (mean, 57%) in relation to the initial concentration of the ZEN was observed in monocultures incubated with *Lactobacillus* sp.

*S. cerevisiae* also demonstrated the ability to reduce the concentration of ZEN, and after 6 h of incubation, a significant decrease of 24–42% (average 34%) to level of 57.64–75.60 μg/ml (mean, 65.55 μg/ml) was observed. In subsequent hours of incubation, the concentration of the mycotoxin further reduced, and after 24 h, ZEN concentrations ranged between 41.88 and 55.84 μg/ml (average reduction of 52% of the initial concentration) (Table 9).

**Table 9:** Reduction of ZEN concentration by *Lactobacillus* and *S. cerevisiae* strains

	Strain	Time (h)			
		0	6	12	24
		Concentration ± SD (μg/ml) (decrease (%))			
*Lactobacillus*	*brevis* ŁOCK 1093	100 ^A^	63.61 ± 3.73 ^B^ (36)	51.71 ± 2.57 ^C^ (48)	43.17 ± 1.36 ^D^ (57)
	*casei* ŁOCK 0911		64.77 ± 3.78 ^B^ (35)	57.41 ± 1.95 ^C^ (43)	49.91 ± 2.33 ^D^ (50)
	*casei* ŁOCK 0915		63.69 ± 2.31 ^B^ (36)	55.07 ± 1.82 ^C^ (45)	51.22 ± 2.54 ^C^ (49)
	*paracasei* ŁOCK 1091		55.86 ± 2.35 ^B^ (44)	50.67 ± 2.33 ^B^ (49)	45.97 ± 1.38 ^C^ (54)
	*pentosus* ŁOCK 1094		41.21 ± 1.90 ^B^ (59)	36.26 ± 3.88 ^B^ (63)	32.22 ± 1.47 ^B^ (67)
	*plantarum* ŁOCK 0860		44.36 ± 1.29 ^B^ (56)	35.42 ± 0.93 ^C^ (65)	27.39 ± 1.46 ^D^ (73)
	*plantarum* ŁOCK 0862		69.93 ± 1.29 ^B^ (30)	62.47 ± 1.73 ^C^ (38)	56.63 ± 1.21 ^D^ (43)
	*reuteri* ŁOCK 1092		47.48 ± 1.04 ^B^ (53)	38.26 ± 0.81 ^C^ (62)	32.84 ± 1.25 ^D^ (67)
	*reuteri* ŁOCK 1096		72.45 ± 3.98 ^B^ (28)	65.37 ± 2.31 ^B^ (35)	60.06 ± 0.64 ^C^ (40)
	*rhamnosus* ŁOCK 1087		60.66 ± 1.78 ^B^ (39)	52.93 ± 2.36 ^C^ (47)	45.64 ± 1.54 ^D^ (54)
	*rhamnosus* ŁOCK 1088		58.00 ± 3.42 ^B^ (42)	48.56 ± 0.65 ^C^ (51)	41.25 ± 1.57 ^D^ (58)
	*rhamnosus* ŁOCK 1089		45.17 ± 1.70 ^B^ (55)	35.05 ± 1.38 ^C^ (65)	30.84 ± 0.82 ^D^ (69)
	Average concentration (μg/ml) (decrease (%))		57.27 (43)	49.10 (51)	43.10 (57)
*S. cerevisiae*	ŁOCK 0068	100 ^A^	75.60 ± 2.88 ^B^ (24)	55.78 ± 2.10 ^C^ (44)	49.99 ± 1.25 ^D^ (50)
	ŁOCK 0113		68.47 ± 1.52 ^B^ (32)	62.14 ± 1.80 ^C^ (38)	55.84 ± 2.72 ^D^ (44)
	ŁOCK 0119		57.64 ± 1.67 ^B^ (42)	47.16 ± 1.61 ^C^ (53)	41.88 ± 1.21 ^D^ (58)
	ŁOCK 0137		58.95 ± 1.68 ^B^ (41)	50.70 ± 2.14 ^C^ (49)	47.47 ± 1.18 ^C^ (53)
	ŁOCK 0140		68.24 ± 2.23 ^B^ (32)	55.59 ± 3.32 ^C^ (44)	47.59 ± 1.31 ^D^ (52)
	ŁOCK 0142		64.38 ± 0.80 ^B^ (36)	52.35 ± 2.83 ^C^ (48)	46.03 ± 0.26 ^D^ (54)
	Average concentration (μg/ml) (decrease (%))		65.55 (34)	53.95 (46)	48.13 (52)

*Values labelled by different capital letters were significantly different per analysed strain (*p* < 0.05)

Based on these results, we concluded that mycotoxin (AFB_1_, DON, FUM, T-2, ZEN) detoxification properties of *Lactobacillus* sp. and *S. cerevisiae* were strain-dependent. Both bacteria and yeast strains, subjected to analysis, showed the highest detoxification activity towards FUM. The most resistant mycotoxin was DON.

We found that the detoxification of AFB_1_, DON, FUM and ZEN mycotoxins by the tested *Lactobacillus* sp. and *S. cerevisiae* strains were similar and did not show significant differences. In contrast, the T-2 compound was more susceptible to removal from the mixture by yeast monocultures at a significance level of *p* < 0.05 compared to bacterial monocultures.

The analysis allowed selection of four bacterial strains and two yeast strains characterised by the best detoxification capabilities of all analysed mycotoxins. These include *Lact. rhamnosus* ŁOCK 1087, *Lact. reuteri* ŁOCK 1092, *Lact. plantarum* ŁOCK 0860, *Lact. pentosus* ŁOCK 1094, *S. cerevisiae* ŁOCK 0119 and *S. cerevisiae* ŁOCK 1042.

## Discussion

Mycotoxin detoxification methods can be classified by modes of action applied before or after harvesting raw plant materials, which are used for human and animal nutrition. During tillage process of plants, good agricultural practice (GAP) should be maintained, including crop rotation, cultivation of resistant plants, ploughing, irrigation, chemical and biological control of plant diseases and proper use of chemicals (e.g. fungicides) [22, 34, 35]. When crops have already been harvested, mycotoxin concentration can be reduced by adjustment of appropriate storage conditions (i.e. humidity and temperature), or by using detoxification treatments (physical, chemical, biological) that can degrade, inactivate or decrease the toxicity level of mycotoxins and ensure the nutritional value of food. Simultaneously, these detoxification methods should not introduce any major changes in production process technology [9, 34, 35].

Among other mycotoxin detoxification methods, microorganisms, inter alia probiotic strains of *Lactobacillus* sp. and *S. cerevisiae*, are used in case of mycotoxin contamination of food and fodder [36]. *Lactobacillus* sp. are able to bind mycotoxins mostly to cell wall peptidoglycans, polysaccharides and teichoic acid, primarily through hydrophobic interactions, whereas *S. cerevisiae* are bind toxic metabolites of filamentous fungi to the cell wall. In addition, microorganisms biodegrade mycotoxins that prevent adsorption of these components inside the intestines on animals that feed on the food [24, 37, 38].

In this article, in vitro results demonstrated the ability to reduce the concentration of mycotoxins: AFB_1_, DON, FUM, T-2, ZEN in PBS solution by 12 strains of bacteria from *Lactobacillus* genus, and 6 *S. cerevisiae* strains.

The AFB_1_ concentration in PBS solution was reduced by the tested strains of bacteria to varying degrees, on average 49.44% after the first 6 h of incubation, by another 4.82% during the next 6 h of incubation, and maintained within a range of 44.20–71.04 μg/ml (average reduction of 59.97%) after 24 h of incubation. The greatest amount of AFB_1_ was bound by bacteria strains after 6 h of incubation, suggesting that the adsorption of the mycotoxin by *Lactobacillus* is an immediate process. Haskard et al. (2001) tested the ability of eight *Lactobacillus* strains to bind AFB_1_ to bacterial surfaces using ELISA; their results were similar to ours [39].

Liew et al. (2018), who also performed AFB_1_ binding assays with ELISA, confirmed results obtained by Haskard et al. (2001), and observed that live cells of *Lactobacillus casei* Shirota were more efficient in binding mycotoxin, than heat-treated organisms [40]. Hernandez-Mendoza et al. (2009), Huang et al. (2017) and Kumar et al. (2018) in their in vitro analysis using HPLC also showed the variable range of detoxification level of AFB_1_ by *Lactobacillus* sp. In these studies, AFB_1_ was bound by the tested strains of bacteria in the range of 14–49%, 20.88–59.44% and 0–85%, respectively [41–43]. The variable binding ability of the *Lactobacillus* strains to AFB_1_ could be the result of differences in cell wall structure, especially in terms of teichoic acid and peptidoglycan content [44]. On the basis of the studies conducted, Gratz et al. (2005) showed that the binding of AFB_1_ by *Lactobacillus* sp*.* is a rapid process, which was also confirmed in research conducted by Kumar et al.(2018), as well as in our studies [43, 45]. Moreover, tested *S. cerevisiae* strains varied in their reduction of AFB_1_ concentration (on average 57.92% after 6 h of incubation, another 3.07% after 12 h of incubation, with a final 3.69% reduction after 24 h), which is consistent with the results obtained by Pizzolitto et al. (2012a) and Poloni et al. (2017) [46, 47]. Strain-dependent AFB_1_ detoxification, by bacteria and yeast, was also observed in the results of studies by Pizzolitto et al. (2011) [48]. The detoxification activity of yeast strains to reduce the concentration of AFB_1_ was similar to that of the tested *Lactobacillus*, and what is also known in the case of yeast is that the cell wall components are also responsible for the binding of the AFB_1_ mycotoxin [48].

On the basis of the results obtained, we conclude that strains of *Lactobacillus* isolated from the intestinal content of monogastric animals showed higher binding activity towards DON, than did strains derived from plant silages. After 6 h of incubation, DON concentration was reduced by the bacterial strains tested by 5.76–21.64% (mean, 15.70%); after 12 h, it was reduced by an average of another 7.39%; and after 24 h, the concentration of DON was reduced by 19.28–39.37% (mean, 29.55%) of the initial concentration of the mycotoxin. Niederkorn et al. (2007) showed low detoxification activity of 137 *Lactobacillus* strains, which was also observed in the strains used in our study [49]. Similar results were obtained by Franco et al. (2011) and Zou et al. (2012), whereas in a study by García et al. (2018), even though they did not observe DON binding by *Lactobacillus rhamnosus* RC007, based on their results, they concluded that *Lact. rhamnosus* contributed to counteract toxic effect of DON and helped to maintain healthy gastrointestinal tract of pigs [50–52]. In our studies, similarly low levels of activity of *S. cerevisiae* were demonstrated, decreasing the concentration of DON by 18.01%, on average, after 6 h of incubation, by 8.80% after another 6 h and an average of 33.03% of the initial concentration after 24 h of incubation. These results are in line with results obtained by Campagnollo et al. (2015) [53]. Based on these data, a significantly weaker DON binding activity by the analysed strains of microorganisms was also observed in comparison to other mycotoxins, namely AF, ZEN or FUM, as also observed by Niderkorn et al. (2007) and Campagnollo et al. (2015) [49, 53].

The 12 *Lactobacillus* strains analysed reduced FB_1_ and FB_2_ mixture concentrations by the most significant amounts among all mycotoxins tested. The FUM concentrations decreased on average by over 51.23% after 6 h of incubation, and a further 8.61% after 12 h. After 24 h of incubation, the concentrations were maintained at an average level of 29.53 μg/ml (reduction by an average of 70.47% of the initial FUM concentration). The greatest reduction in concentration occurred after 6 h, while the FUM binding process was observed up to 24 h of incubation, which is comparable to the results obtained by Abbès et al. (2016) [54]. Zhao et al. (2016) also showed that the process of FUM binding to the surface of *Lactobacillus* is rapid, as they observed the binding after 1 h of incubation [55]. In fact, Pizzolitto et al. (2012b) found that it was an immediate process, occurring after only a minute of incubation [56]. Deepthi et al. (2016) also reported high FB_1_ removal (61.7%) by *Lact. plantarum* MYS6, after 4 h of incubation; furthermore, they observed that tested strain suppressed fumonisin biosynthesis [57]. In our study, high FUM detoxification levels exhibited by *Lactobacillus* sp., which was dependent on the strain tested and mycotoxin type, coincided with the results obtained by Niderkorn et al. (2007), Abbès et al. (2016) and Zhao et al. (2016) [49, 54, 55]. Moreover, Zhao et al. (2016) also noted that peptidoglycan is primarily responsible for the binding of FUM, whereas teichoic acid has little effect on mycotoxin binding [55]. On the other hand, Martinez Tuppia et al. (2016), showed that FB_1_ detoxification might not only be a matter of adsorption, but also the result of biodegradation to other compounds (e.g. hydrolysed fumonisin B1, HFB_1_); however, this was not observed for all analysed *Lactobacillus* sp. strains in their study [58]. In our research, high and diverse activity of the analysed *S. cerevisiae* strains reducing FUM concentration was also shown, and the results agree with those of Pizzolitto et al. (2012b) [56]. Furthermore, Armando et al. (2013), who also observed high FB_1_ binding properties of *S. cerevisiae* yeast (strain RC016), observed that ability of binding the mycotoxin increases along with concentration [59]. Our results also showed that the strains isolated from the feed were characterised by a slightly lower ability to remove FUM (67.43% after 24 h of incubation) than achieved by baker’s yeast (70.71–74.47% reduction of FUM concentration after 24 h of incubation) or strains isolated from a distillery environment (72.88% and 73.04% reduction of mycotoxin concentration after 24 h of incubation).

The concentrations of T-2 toxin were decreased by the tested strains of *Lactobacillus* and *S. cerevisiae* to a high degree, where reduction exceeded a mean of 60% after 24 h of incubation. After 6 h of incubation, the *Lactobacillus* strains reduced the concentration of T-2 by 26.68–51.75% (average 42.64%), and after 24 h, it remained at a level of 31.09–50.10 μg/ml (average 39.01 μg/ml). Bacterial strains used in our study showed higher T-2 binding affinity than shown by the four strains analysed by Zou et al. (2012) [51]. Only in relation to the T-2 toxin did the six *S. cerevisiae* strains show significantly stronger detoxification properties (average T-2 binding after 24 h of incubation was 69.18%) than the tested bacterial strains (average decontamination rate after 24 h was 61.45%). Zou et al. (2015) obtained a similar level of T-2 detoxification by *S. cerevisiae* strains in their studies and found the yeast’s reduction of the concentration of T-2 toxin was also the result of mycotoxin binding to the yeast cell wall [60].

The ability of *Lactobacillus* strains to bind ZEN was similar to that observed in the case of AFB_1_ and the detoxification level of the mycotoxin was mean of 56.90% after 24 h of incubation. Our results showed that the greatest reduction in ZEN concentrations, by 27.55–58.79% (mean, 42.73%), occurred after 6 h of incubation. The ZEN concentrations were reduced further by an average of 8.17% after the next 6 h of incubation, finally reaching an average level of 43.10 μg/ml after 24 h (an average 56.90% of the initial concentration of ZEN was bound by the bacterial strains). The capability of *Lactobacillus* strains to decrease ZEN concentration was also found by Zhao et al. (2015); ZEN binding activities on the cell walls of the 27 tested *Lact. plantarum* strains were weaker compared to those of the strains we used in our study [61]. Furthermore, Zhao et al. established that the binding of ZEN to the cell wall of *Lactobacillus* is an immediate process, which is consistent with the results we obtained [61]. Niderkorn et al. (2006), Čvek et al. (2012), Sangsila et al. (2016) and Vega et al. (2017) showed comparable results from their studies indicating great potential of *Lactobacillus* sp. to reduce ZEN concentration [62–65]. We found that *S. cerevisiae* strains reduced ZEN concentration by 24.40–42.36% (average 34.45%) after 6 h of incubation, with an additional 11.59% after 12 h and an average 5.82% after 24 h. Similar results were obtained by Keller et al. (2015); they concluded that at the beginning, large amounts of ZEN is bound to the yeast cell wall due to the higher concentration of the mycotoxin in the environment [66]. The ZEN detoxification activity of the bacteria and yeast strains used in this study, was strain-dependent, a result also shown by Armando et al. (2012) and Zhao et al. (2015) [61, 67].

## Conclusions

Our data allowed us to observe the potential of *Lactobacillus* and *S. cerevisiae* to lower the concentrations of the mycotoxins AFB_1_, DON, FUM, T-2 and ZEN. We found that the detoxification of these compounds is a rapid process. To a great extent, the concentration of the toxins decreases within 6 h of incubation, and can last up to 24 h. Nevertheless, the differences in concentrations were not so significant at a prolonged period of 24-h incubation.

The *Lactobacillus* and *S. cerevisiae* strains analysed showed detoxification properties towards mycotoxins (AFB_1_, DON, FUM, T-2, ZEN) in this in vitro study. Therefore, these strains, after further investigation, could be used as food and feed additives to detoxify contaminating mycotoxins, which pose potential threats to human and animal health.

## References

[1] Smith MC, Madec S, Coton E, Hymery N (2016). Natural co-occurrence of mycotoxins in foods and feeds and their *in vitro* combined toxicological effects. Toxins.

[2] Alshannaq A, Yu JH (2017). Occurrence, toxicity, and analysis of major mycotoxins in food. Int J Environ Res Public Health.

[3] Liew WPP, Mohd-Redzwan S (2018) Mycotoxin: its impact on gut health and microbiota. Front Cell Infect Microbiol 8(60). 10.3389/fcimb.2018.00060 10.3389/fcimb.2018.00060 PMC5834427 29535978

[4] Gallo A, Giuberti G, Frisvad JC, Bertuzzi T, Nielsen KF (2015). Review on mycotoxin issues in ruminants: occurrence in forages, effects of mycotoxin ingestion on health status and animal performance and practical strategies to counteract their negative effects. Toxins.

[5] Pinotti L, Ottoboni M, Giromini C, Dell’Orto V, Cheli F (2016). Mycotoxin contamination in the EU feed supply chain: a focus on cereal byproducts. Toxins.

[6] Freire L, Sant’Ana AS (2018). Modified mycotoxins: an updated review on their formation, detection, occurrence, and toxic effects. Food Chem Toxicol.

[7] Streit E, Schatzmayr G, Tassis P, Tzika E, Marin D, Taranu I, Tabacu C, Nicolau A, Aprodu I, Puel O, Oswald IP (2012). Current situation of mycotoxin contamination and co-occurrence in animal feed-focus on Europe. Toxins.

[8] Ji C, Fan Y, Zhao L (2016). Review on biological degradation of mycotoxins. Animal Nutr.

[9] Zacharisova M, Dzuman Z, Veprikova Z, Hajkova K, Jiru M, Vaclavikova M, Zacharisaova A, Pospichalova M, Florian M, Hajslova J (2014). Occurrence of multiple mycotoxins in European feedingstuffs, assessment of dietary intake by farm animals. Anim Feed Sci Technol.

[10] Neme K, Mohammed A (2017). Mycotoxin occurrence in grains and the role of postharvest management as a mitigation strategies. A review. Food Control.

[11] Kosicki R, Błajet-Kosicka A, Grajewski J, Twarużek M (2016). Multiannual mycotoxin survey in feed materials and feedingstuffs. Anim Feed Sci Technol.

[12] European Parliament and the Council (2002) Directive 2002/32/EC of the European Parliament and of the Council of 7 May 2002 on undesirable substances in animal feed - Council statement. https://eur-lex.europa.eu/resource.html?uri=cellar:aca28b8c-bf9d-444f-b470-268f71df28fb.0004.02/DOC_1&format=PDF. **Accessed 06 April 2018**

[13] The Commission Of The European Communities (2006) Commission Regulation (EC) No 1881/2006 of 19 December 2006 setting maximum levels for certain contaminants in foodstuffs. http://eur-lex.europa.eu/legal-content/EN/TXT/PDF/?uri=CELEX:32006R1881&from=en. **Accessed 06 April 2018**

[14] The Commission Of The European Communities (2006) Commission recommendation 2006/576/EC of 17 August 2006 on the presence of deoxynivalenol, zearalenone, ochratoxin A, T-2 and HT-2 and fumonisins in products intended for animal feeding. http://eur-lex.europa.eu/legal-content/EN/TXT/PDF/?uri=CELEX:32006H0576&from=EN. **Accessed 06 April 2018**

[15] Medina A, Magan N (2011). Temperature and water activity effects on production of T-2 and HT-2 by *Fusarium langsethiae* strains from north European countries. Food Microbiol.

[16] Milani JM (2013). Ecological conditions affecting mycotoxin production in cereals: a review. Vet Med (Praha).

[17] The Commission Of The European Communities (2013) Commission Recommendation 2013/165/EU of 27 March 2013 on the presence of t-2 and ht-2 toxin in cereals and cereal products. http://eur-lex.europa.eu/legal-content/EN/TXT/PDF/?uri=CELEX:32013H0165&from=EN. **Accessed 06 April 2018**

[18] Nugmanov A, Beishova I, Kokanov S, Lozowicka B, Kaczynski P, Konecki R, Snarska K, Wołejko E, Sarsembayeva N, Abdigaliyeva T (2018). Systems to reduce mycotoxin contamination of cereals in the agricultural region of Poland and Kazakhstan. Crop Prot.

[19] The European Parliament And The Council Of The European Union (2017) Regulation (EU) 2017/625 of the European Parliament and of the Council of 15 March 2017 on official controls and other official activities performed to ensure the application of food and feed law, rules on animal health and welfare, plant health and plant protection products. https://eur-lex.europa.eu/legal-content/EN/TXT/PDF/?uri=CELEX:32017R0625&from=EN. **Accessed 06 April 2018**

[20] Aiko V, Mehta L (2015). Occurrence, detection and detoxification of mycotoxins. J Biosci.

[21] Loi M, Fanelli F, Liuzzi VC, Logrieco AF, Mulè G (2017). Mycotoxin biotransformation by native and commercial enzymes: present and future perspectives. Toxins.

[22] Zhu Y, Hassan YI, Lepp D, Shao S, Zhou T (2017). Strategies and methodologies for developing microbial detoxification systems to mitigate mycotoxins. Toxins.

[23] Juodeikiene G, Bartkiene E, Cernauskas D, Cizeikiene D, Zadeike D, Lele V, Bartkevics V (2018). Antifungal activity of lactic acid bacteria and their application for *Fusarium* mycotoxin reduction in malting wheat grains. LWT-Food Sci Technol.

[24] El-Nezami H, Kankaanpaa P, Salminen S, Ahokas J (1998). Ability of dairy strains of lactic acid bacteria to bind a common food carcinogen, aflatoxin B1. Food Chem Toxicol.

[25] FAO/WHO (2002) Guidelines for the evaluation of probiotics in food. **Food and Agriculture Organization of the United Nations and World Health Organization Working Group Report**. http://www.fao.org/3/a-a0512e.pdf. Accessed 24 March 2018

[26] Vinderola G, Ritieni A (2015). Role of probiotics against mycotoxins and their deleterious effects. J Food Res.

[27] Śliżewska K, Chlebicz A (2017) Strains of lactic acid bacteria from the genus *Lactobacillus*. Patent Application no 422603

[28] Aflatoxin B_1_ from *Aspergillus flavus*, Sigma-Aldrich. https://www.sigmaaldrich.com/catalog/product/sigma/a6636?lang=pl&region=PL. Accessed 21 March 2018

[29] Deoxynivalenol, Sigma-Aldrich. https://www.sigmaaldrich.com/catalog/product/sial/32943?lang=pl&region=PL. Accessed 21 March 2018

[30] Fumonisin B_1_, Sigma-Aldrich. https://www.sigmaaldrich.com/catalog/product/sial/34139?lang=pl&region=PL&cm_sp=Insite-_-prodRecCold_xorders-_-prodRecCold2-1. Accessed 21 March 2018

[31] Fumonisin B_2_, Sigma-Aldrich. https://www.sigmaaldrich.com/catalog/product/sial/34142?lang=pl&region=PL&cm_sp=Insite-_-prodRecCold_xorders-_-prodRecCold2-2. Accessed 21 March 2018

[32] T-2 toxin, Sigma-Aldrich. https://www.sigmaaldrich.com/catalog/product/sigma/t4887?lang=pl&region=PL. Accessed 21 March 2018

[33] Zearalenone, Sigma-Aldrich. https://www.sigmaaldrich.com/catalog/product/sigma/z2125?lang=pl&region=PL. Accessed 21 March 2018

[34] Devreese M, De Backer P, Croubels S (2013). Different methods to counteract mycotoxin production and its impact on animal health. Vlaams Diergeneeskd Tijdschr.

[35] Karlovsky P, Suman M, Berthiller F, De Meester J, Eisenbrand G, Perrin I, Oswald IPm Speijers G, Chiodini A, Recker T, Dussort P (2016). Impact of food processing and detoxification treatments on mycotoxin contamination. Mycotoxin Res.

[36] Moslehi-Jenabian S, Pedersen LL, Jespesen L (2010). Beneficial effects of probiotic and food borne yeasts on human health. Nutrients.

[37] Śliżewska K, Smulikowska S (2011). Detoxification of aflatoxin B_1_ and change in microflora pattern by probiotic in vitro fermentation of broiler feed. Anim Feed Sci Technol.

[38] Vila-Donat P, Marin S, Sachis V, Ramos AJ (2018). A review of the mycotoxin adsorbing agents, with an emphasis on their multi-binding capacity, for animal feed decontamination. Anim Feed Sci Technol.

[39] Haskard CA, El-Nezmi HS, Kankaanpää PE, Salminen S, Ahokas JT (2001). Surface binding of aflatoxin B_1_ by lactic acid bacteria. Appl Environ Microbiol.

[40] Liew WPP, Nurul-Adilah Z, Than LTL, Mohd-Redzwan S (2018) The binding efficiency and interaction of *Lactobacillus casei* Shirota toward aflatoxin B_1_. Front Microbiol 9(1503). 10.3389/fmicb.2018.01503 10.3389/fmicb.2018.01503 PMC6048233 30042748

[41] Hernandez-Mendoza A, Garcia HS, Steele JL (2009). Screening of *Lactobacillus casei* strains for their ability to bind aflatoxin B_1_. Food Chem Toxicol.

[42] Huang L, Duan C, Zhao Y, Gao L, Niu C, Xu J, Li S (2017). Reduction of aflatoxin b_1_ toxicity by lactobacillus plantarum c88: a potential probiotic strain isolated from Chinese traditional fermented food “tofu”. PLoS One.

[43] Kumar SS, Bashisht A, Venkateswaran G, Hariprasad P, Gayathri D (2018) Characterization of novel *Lactobacillus fermentum* from curd samples of indigenous cows from Malnad region, Karnataka, for their aflatoxin B_1_ binding and probiotic properties. Probiotics Antimicrob Proteins. 10.1007/s12602-018-9479-7 10.1007/s12602-018-9479-7 30368716

[44] Hernandez-Mendoza A, Guzman-de-Peña D, Garcia HS (2009) Key role of 383 teichoic acids on aflatoxin B_1_ 384 binding by probiotic bacteria. J Appl Microbiol 107:395–403. 10.1111/j.1365-3852672.2009.04217.x 10.1111/j.1365-2672.2009.04217.x 19486416

[45] Gratz S, Mykkänen H, El-Nezami H (2005). Aflatoxin B_1_ binding by a mixture of *Lactobacillus* and *Propionibacterium*: *in vitro* versus *ex vivo*. J Food Prot.

[46] Pizzolitto RP, Armando MR, Combina M, Cavaglieri LR, Dalcero AM, Salvano MA (2012). Evaluation of *Saccharomyces cerevisiae* strains as probiotic agent with aflatoxin B_1_ adsorption ability for use in poultry feedstuffs. J Environ Sci Health B.

[47] Poloni V, Salvato L, Pereyra L, Oliveira A, Rosa C, Cavaglieri L, Keller KM (2017). Bakery by-products based feeds borne-*Saccharomyces cerevisiae* strains with probiotic and antimycotoxin effects plus antibiotic resistance properties for use in animal production. Food Chem Toxicol.

[48] Pizzolitto RP, Bueno DJ, Armando MR, Cavaglieri L, Dalcero AM, Salvano MA (2011) Binding of aflatoxin B_1_ to lactic acid bacteria and *Saccharomyces cerevisiae in vitro*: a useful model to determine the most efficient microorganism. In: Guevara-Gonzalez RG (ed) Aflatoxins. Biochemistry and molecular biology, InTech. 10.5772/23717. https://www.intechopen.com/books/aflatoxins-biochemistry-and-molecular-biology/binding-of-aflatoxin-b1-to-lactic-acid-bacteria-and-saccharomyces-cerevisiae-in-vitro-a-useful-model. **Accessed 28 March 2018**

[49] Niderkorn V, Morgavi DP, Pujos E, Tissandier A, Boudra H (2007). Screening of fermentative bacteria for their ability to bind and biotransform deoxynivalenol, zearalenone and fumonisins in an *in vitro* simulated corn silage model. Food Addit Contam.

[50] Franco TS, Garcia S, Hirooka EY, Ono YS, Santos JS (2011). Lactic acid bacteria in the inhibition of *Fusarium graminearum* and deoxynivalenol detoxification. J Appl Microbiol.

[51] Zou ZY, He ZF, Li HJ, Han PF, Meng X, Zhang Y, Zhou F, Ouyang KP, Chen XY, Tang J (2012). *In vitro* removal of deoxynivalenol and T-2 toxin by lactic acid bacteria. Food Sci Biotechnol.

[52] García GR, Payros D, Pinton P, Dogi CA, Laffitte J, Neves M, González Pereyra ML, Cavaglieri LR, Oswald IP (2018). Intestinal toxicity of deoxynivalenol is limited by *Lactobacillus rhamnosus* RC007 in pig jejunum explants. Arch Toxicol.

[53] Campagnollo FB, Franco LT, Rottinghaus GE, Kobashigawa E, Ledoux DR, Daković A, Oliveira CAF (2015). *In vitro* evaluation of the ability of beer fermentation residue containing *Saccharomyces cerevisiae* to bind mycotoxins. Food Res Int.

[54] Abbès S, Salah-Abbès JB, Jebali R, Younes RB, Oueslati R (2016). Interaction of aflatoxin B_1_ and fumonisin B_1_ in mice causes immunotoxicity and oxidative stress: possible protective role using lactic acid bacteria. J Immunotoxicol.

[55] Zhao H, Wang X, Zhang J, Zhang J, Zhang B (2016). The mechanism of *Lactobacillus* strains for their ability to remove fumonisins B_1_ and B_2_. Food Chem Toxicol.

[56] Pizzolitto RP, Salvano MA, Dalcero AM (2012). Analysis of fumonisin B_1_ removal by microorganisms in co-occurrence with aflatoxin B_1_ and the nature of the binding process. Int J Food Microbiol.

[57] Deepthi BV, Poornachandra Rao K, Chennapa G, Naik MK, Chandrashekara KT, Sreenivasa MY (2016). Antifungal attributes of *Lactobacillus plantarum* MYS6 against fumonisin producing *Fusarium proliferatum* associated with poultry feeds. PLoS One.

[58] Martinez Tuppia C, Atanasova-Penichon V, Chéreau S, Ferrer N, Marchegay G, Savoie J, Richard-Forget F (2017). Yeast and bacteria from ensiled high moisture maize grains as potential mitigation agents of fumonisin B_1_. J Sci Food Agric.

[59] Armando M, Galvagno M, Dogi C, Cerrutti P, Dalcero A, Cavaglieri L (2013). Statistical optimization of culture conditions for biomass production of probiotic gut-borne *Saccharomyces cerevisiae* strain able to reduce fumonisin B_1_. J Appl Microbiol.

[60] Zou Z, Sun J, Huang F, Feng Z, Li M, Shi R, Ding J, Li H (2015). *In vitro* removal of T-2 toxin by yeasts. J Food Safety.

[61] Zhao L, Jin H, Zhang R, Ren H, Zhang X, Yu G (2015). Detoxification of zearalenone by three strains of *Lactobacillus plantarum* from fermented food in vitro. Food Control.

[62] Niderkorn V, Boudra H, Morgavi DP (2006). Binding of *Fusarium* mycotoxins by fermentative bacteria *in vitro*. J Appl Microbiol.

[63] Čvek D, Markov K, Frece J, Friganović M, Duraković L, Delaš F (2012). Adhesion of zearalenone to the surface of lactic acid bacteria cells. Croat J Food Tech Biotech Nutr.

[64] Sangsila A, Faucet-Marquis V, Pfohl-Leszkowicz A, Itsaranuwat P (2016). Detoxification of zearalenone by *Lactobacillus pentosus* strains. Food Control.

[65] Vega MF, Dieguez SN, Riccio B, Aranguren S, Giordano A, Denzoin L, Soraci AL, Tapia MO, Ross R, Apás A, González SN (2017). Zearalenone adsorption capacity of lactic acid bacteria isolated from pigs. Braz J Microbiol.

[66] Keller L, Abrunhosa L, Keller K, Rosa CA, Cavaglieri L, Venâncio A (2015). Zearalenone and its derivatives α-zearalenol and β-zearalenol decontamination by *Saccharomyces cerevisiae* strains isolated from bovine forage. Toxins.

[67] Armando MR, Pizzolitto RP, Dogi CA, Cristofolini A, Merkis C, Poloni V, Dalcero AM, Cavaglieri LR (2012). Adsorption of ochratoxin A and zearalenone by potential probiotic *Saccharomyces cerevisiae* strains and its relations with cell wall thickness. J Appl Microbiol.

